# Long-term gait measurements in daily life: Results from the Berlin Aging Study II (BASE-II)

**DOI:** 10.1371/journal.pone.0225026

**Published:** 2019-12-11

**Authors:** Jörn Kiselev, Timur Nuritdinow, Dominik Spira, Nikolaus Buchmann, Elisabeth Steinhagen-Thiessen, Christian Lederer, Martin Daumer, Ilja Demuth

**Affiliations:** 1 Geriatrics Research Group, Charité–Universitätsmedizin Berlin, corporate member of Freie Universität Berlin, Humboldt-Universität zu Berlin, and Berlin Institute of Health, Berlin, Germany; 2 Department of Anesthesiology and Intensive Care Medicine, Campus Charité Mitte, corporate member of Freie Universität Berlin, Humboldt-Universität zu Berlin, and Berlin Institute of Health, Berlin, Germany; 3 Sylvia Lawry Centre for Multiple Sclerosis Research e.V., The Human Motion Institute, Munich, Germany; 4 Lipid Clinic at the Interdisciplinary Metabolism Center, Charité–Universitätsmedizin Berlin, corporate member of Freie Universität Berlin, Humboldt-Universität zu Berlin, and Berlin Institute of Health, Berlin, Germany; 5 Department of Cardiology, Campus Benjamin Franklin, Charité—University Medicine Berlin, corporate member of Freie Universität Berlin, Humboldt-Universität zu Berlin, and Berlin Institute of Health, Berlin, Germany; 6 Trium Analysis Online GmbH, Munich, Germany; 7 Charité—Universitätsmedizin Berlin, BCRT—Berlin Institute of Health Center for Regenerative Therapies, Berlin, Germany; University of Malaya, MALAYSIA

## Abstract

**Background:**

Walking ability is an important prerequisite for activity, social participation and independent living. While in most healthy adults, this ability can be assumed as given, limitations in walking ability occur with increasing age. Furthermore, slow walking speed is linked to several chronic conditions and overall morbidity. Measurements of gait parameters can be used as a proxy to detect functional decline and onset of chronic conditions. Up to now, gait characteristics used for this purpose are measured in standardized laboratory settings. There is some evidence, however, that long-term measurements of gait parameters in the living environment have some advantages over short-term laboratory measurements.

**Methods:**

We evaluated cross-sectional data from an accelerometric sensor worn in a subgroup of 554 participants of the Berlin Aging Study II (BASE-II). Data from the two BASE-II age groups (age between 22–36 years and 60–79 years) were used for the current analysis of accelerometric data for a minimum of two days and a maximum of ten days were available. Real world walking speed, number of steps, maximum coherent distance and total distance were derived as average data per day. Linear regression analyses were performed on the different gait parameters in order to identify significant determinants. Additionally, Mann-Whitney-U-tests were performed to detect sex-specific differences.

**Results:**

Age showed to be significantly associated with real world walking speed and with the total distance covered per day, while BMI contributed negatively to the number of walking steps, maximum coherent distance and total distance walked. Additionally, sex was associated with walking steps. However, R^2^-values for all models were low. Overall, women had significantly more walking steps and a larger coherent distance per day when compared to men. When separated by age group, this difference was significant only in the older participants. Additionally, walking speed was significantly higher in women compared to men in the subgroup of older people.

**Conclusions:**

**A**ge- and sex-specific differences have to be considered when objective gait parameters are measured, e.g. in the context of clinical risk assessment. For this purpose normative data, differentiating for age and sex would have to be established to allow reliable classification of long-term measurements of gait.

## Introduction

The ability to walk is a very important one especially for older people, as it not only allows them to continue any type of social participation that requires changing locations (e.g. meeting friends, visiting theater or cinema) but also is an important factor to stay active and healthy. Walking ability can be compromised by many factors such as joint disorders, muscle weakness, sensomotoric deficits or pain [1]. Functional parameters including walking speed [WS] or cadence are frequently used to measure walking ability. WS measurements are quick and easy to perform and have been demonstrated to have a good validity with respect to prediction of a decline in mobility and high levels of reliability [2,3]. Cesari et al. (2005) conducted a study with 3.047 well-functioning older adults in which a usual WS of less than 1 m/sec as a cut-off point was associated with a high risk of developing functional limitations of the lower extremities, hospitalization and death within the next year [4]. In a systematic review on WS in geriatric clinical settings, an average speed of 0.58 m/sec at usual walking speed and 0.89 m/sec at maximum speed could be calculated, demonstrating the mobility limitations of geriatric patients with medical issues in need of medical intervention [5].

Lower levels of WS have shown to be associated with several chronic conditions such as diabetes type 2 hypertension or past fractures [6], and functional status in COPD [7], conditions typically to be found in older people.

Additionally, in a systematic review by Pamoukdjian et al. (2015), WS was identified as an independent factor for the identification of older people at risk of frailty as well as an overall predictor for sarcopenia, disability in activities of daily living (ADL), falls, hospitalization or institutionalization and mortality [8]. This was confirmed in a systematic review by Clegg et al. (2015) [9]. In this review, a walking speed of <0.8 m/sec resulted in a 0.99 sensitivity and a 0.64 specificity for identifying older people with frailty. In another systematic review, slow WS was associated with overall mortality too [10], while in a large population-based cohort study, overall mortality could be predicted by a walking cadence of less than 100 steps per minute [11]. In summary, as described in a comprehensive review of the literature by Abellan van Kan et al. (2009), WS was found to be a consistent risk factor for disability, cognitive impairment, institutionalization, falls and mortality [12].

While these findings demonstrate the clinical importance of being able to walk at a reasonable speed, a decline in WS has direct consequences for older people apart from health-related factors. Two UK-based studies exhibited that the majority of two large cohorts of older people were not able to cross a road safely [13,14]. Thus, it is not surprising that in a cohort of 3,070 older people, slow walking speed and social isolation were found to be associated [15].

WS and cadence can be measured in several ways. The simplest way to measure WS is to mark a distance on the floor and use a stopwatch to stop the time needed to cover that distance and to count the number of steps taken. From this, mean walking speed and cadence can be derived. Goldberg et al. (2011) could demonstrate the validity and minimal detectable change (MDC) of a 4-meter walking speed test using a stopwatch [16]. However, longer measurement distances might be able to produce more stable results [17].

A recent publication by Brodie et al (2017) was able to demonstrate that gait characteristics measured in daily life were able to differentiate between fallers and non-fallers while measurements in a clinical setting were not able to provide this information [18]. This suggests that walking patterns are influenced by the environment and setting in which measurements take place. Based on this work, one can argue that measurements in the living environment of participants over a longer period of time produces more relevant data and thus should be preferred to laboratory measurements when applicable. Such measurements require the use of inertial accelerometric sensors for a continuous capturing of gait characteristics. Additionally, when using such a measurement protocol, the question arises if established cut-off points for WS can still be used for interpreting results from long-term measurements of walking speed in a real-life environment (Real-World Walking Speed, RWWS). Therefore, basic research is needed to establish normative data on RWWS as well as other gait parameters for different target populations.

In a publication by Schimpl et al. (2011) [19], a mobile accelerometric system was used to evaluate the association between walking speed and age in a cohort of middle-aged adults from the UK who were wearing the accelerometric device for seven consecutive days. Based on this work, a first data set consisting of RWWS, running steps and total distance per day was presented to demonstrate the external validity of the measurement system. However, as the cohort in this study consisted of UK-based blood donors with a wide range of age, data on older adults as one of several subgroups were limited. Therefore, we conducted a data analysis of a sub-cohort of the Berlin Aging Study II (BASE-II) using the same sensor as Schimpl and colleagues. The aim of this analysis was to present data on younger and older adults from a German cohort using the same measurement approach and to compare these data to those presented by Schimpl et al. (2011) [19]. We hypothesize therefore that our analysis of RWWS in two groups of younger and older adults would confirm the findings of Schimpl et al. and thus provide a broader basis for the use of RWWS as a tool for measuring WS and factors associated with changes in RWWS in older people.

## Materials and methods

The BASE-II was launched to investigate factors associated with “healthy” or “unhealthy” aging. The cohort was drawn as a convenience sample from the greater metropolitan area of Berlin, Germany [20,21]. 2,172 participants in two age groups (~75% aged 60–84 years and ~25% aged 20–35 years) were recruited for the medical part of the study. All participants gave written informed consent and the Ethics Committee of the Charité—Universitätsmedizin Berlin approved the study (approval number EA2/029/09).

A subgroup of 593 participants from the BASE-II study was equipped with a 3-dimensional accelerometer (actibelt, Trium Analysis Online, Munich; Germany) for a minimum of seven consecutive days. As this procedure was part of the standard study protocol, all participants received written and oral information about the accelerometric measurements as part of the participation in the main study.

### Actibelt tri-axial accelerometer

The actibelt is a tri-axial accelerometer worn near the center of mass of the user with the help of a belt. The device has a sampling frequency of 100Hz. Battery and memory capacity within the actibelt RCT1, the model used in this study, allow for continuous measurements of up to 10 days [22]. The trained support vector machine for speed estimation of the actibelt sensor was successfully validated for the use in populations of healthy individuals in a free-living environment using a measurement wheel with a mounted speedometer as reference standard [23]. The measurement wheel provided precise distances and gait speeds for different walking procedures ranging from deliberately slow walking to running. The results from the reference standard were the compared with the data of the actibelt accelerometer. For this, step lengths and number of steps measured with the accelerometer were multiplied to derive measured distances; furthermore, gait velocity was calculated from these data. To compare these measurements with the reference data, coverage probability (CP) and Lin’s concordance correlation coefficient (CCC) were calculated. The CP is the proportion of measurements containing the “true” value of the reference standard while the CCC compares a bivariate set of data to a reference standard [24]. With the CP should not be lower than 1-α, the CCC can show values between -1 and +1, with +1 demonstrating “perfect correlation” [25]. In the study by Schimpl et al. (2001, the CP was 0.99 (0.84–1.0) and the CCC was 00.97 (0.97–0.98) in a model with a maximum difference of 0.3 m/sec for measured gait speeds. In another study, the actibelt demonstrated a high level of measurement accuracy in patients with multiple sclerosis [26] during the 6-Minute-walk-test (6MWT), although a slight overestimation of 0.12 to 0.16 m/sec could be observed in patients with higher levels of disability.

In the BASE-II study, the actibelt sensor monitored the time the sensor was worn and active, time spent in low, medium and high level physical activity, the number of walking and running steps, distance covered walking and running, maximum coherent distance covered, and average speed in walking and running for each day the belt was worn. Additionally, the average speed during events in which participants covered at least 50 and 100 meters were calculated for each measurement day. From these data, average values over all measurement days, in which the belt was active and worn for at least 10 hours, were calculated.

## Data analysis

Sex, age, height, and BMI were derived from the participant file of the main study for all participants wearing the actibelt system. Participants with no available socio-demographic data were excluded from the analysis. Furthermore, to be considered for the analysis, participants had to wear the actibelt for at least 2 days for a minimum of 10 hours.

### Statistical analysis

Statistical analysis was carried out using the software package SPSS 25 for Windows (IBM Inc., Chicago, USA). All interval-scaled data were tested for normal distribution using the Kolmogorov-Smirnov (KS)-test. Based on the results of this test, either mean scores with standard deviations (SD) or median values with interquartile ranges (IQR) are reported. For comparing age-related effects on gait, the entire cohort as well as the subgroups of younger and older adults was analyzed. Additionally, we performed the Mann-Whitney-U-Test for testing significant differences between men and women regarding the analyzed gait parameters including a Bonferroni correction for multiple testing. Furthermore, a multiple linear regression analysis was executed for each parameter to test the predictability of these parameters from age, height, sex and BMI. As part of this regression analysis, an outlier analysis was performed resulting in the exclusion of four data sets with values beyond the 3-fold SD of the values of all participants. All statistical tests were performed with a α-level of 0.05 and β = 0.8 unless adapted due to multiple testing.

## Results

**Fig 1:**
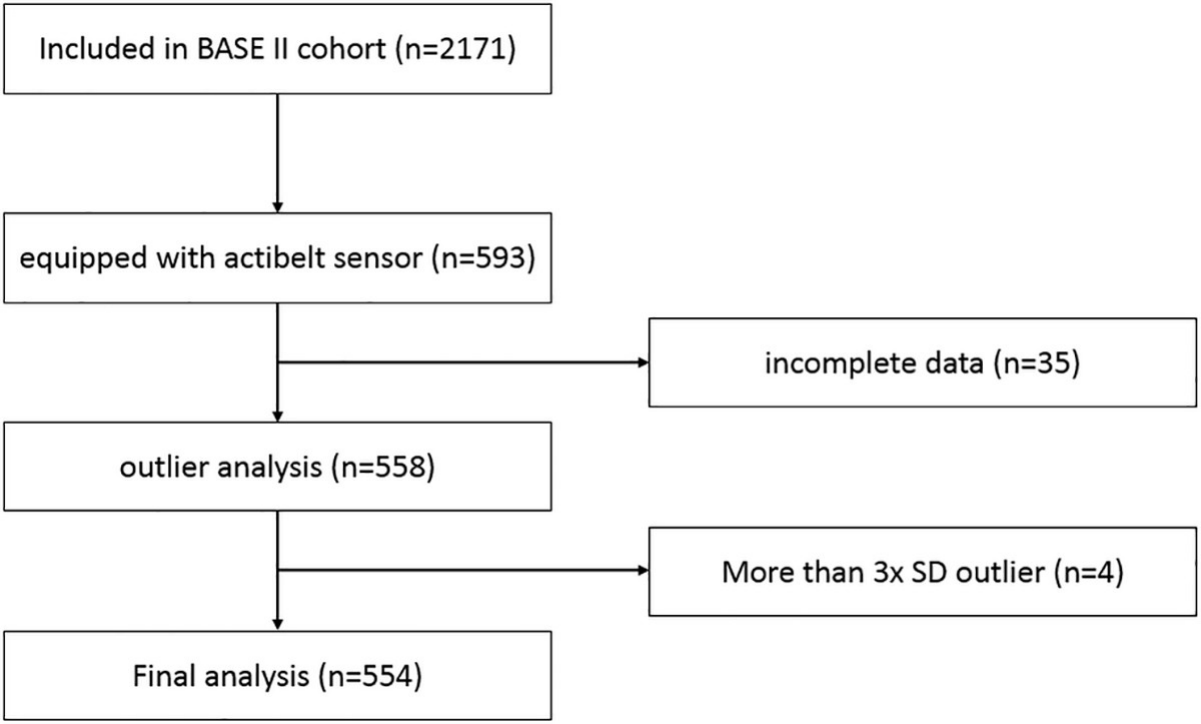
Flow diagram of participant inclusion.

554 participants were included into the final analysis, as can be seen in Fig 1. The median of measurement days was seven days with a range of two to eleven days (IQR 2). 60.7% of the participants were male and 39.2% were female. Age, height and BMI were not normally distributed; therefore, median and IQR values are presented in Table 1.

**Table 1:** Sociodemographic variables (n = 554).

Variable	All participants		Younger participants		Older participants	
	N	%	N	%	N	%
**Sex**						
*Male*	337	60.8	67	56.8	270	60.8
*Female*	217	39.2	51	43.2	166	39.2
	**Median**	**IQR (Range)**	**Median**	**IQR (Range)**	**Median**	**IQR (Range)**
**Age (years)**						
*All*	67	7 (22–79)	28	5 (22–36)	69	6 (60–79)
*Male*	67	7 (22–79)	28	3 (22–36)	68	6 (60–80)
*Female*	67	9 (22–76)	28	6 (22–36)	69	5 (61–76)
Height (cm)*						
*All*	172.8	12.8 (149.2–199.7)	176.2	42.0 (157.7–199.7)	171.7	13.05 (149.2–197.0)
*Male*	176.4	8.3 (161.5–199.7)	180.1	9.1 (165.1–199.7)	175.7	7.83 (161.5–197.0)
*Female*	163.7	9.6 (149.2–187.0)	170.0	11.4 (157.7–187.0)	162.6	7.83 (149.2–181.0)
BMI*						
*All*	25.8	5.2 (17.0–43.5)	23.14	4.76 (17.0–40.5)	26.37	4.92 (17.7–43.5)
*Male*	26.2	4.7 (17.8–42.6)	23.92	4.28 17.8–30.5)	26.71	4.24 (20.0–42.6)
*Female*	24.5	5.59 (17–43.5)	21.51	5.14 (17.0–40.)	25.33	5.25 (17.7–43.5)

Abr.: BMI: Body Mass Index; IQR: Interquartile Range; N: number of participants

* Significant differences between male and female participants (p<0.001)

For all participants as well as for both age groups, no significant differences were found for age between both sexes. However, both height and BMI were significantly higher in males when compared to female participants (p<0.001). When comparing sociodemographic data of the study cohort with the total study population of the BASE II cohort, comparable distributions of all variables could be obtained with the exception of the proportion of male and female participants (see Table 1, supporting information).

Table 2 shows the results of the accelerometric measurements. Average data over all included measurement days on the number of walking steps, maximum coherent distance walked, total distance walked and gait speed while walking are shown.

**Table 2:** Results of walking parameters (n = 554).

Variable	All participants		Younger participants		Older participants	
	Median	IQR (Range)	Median	IQR (Range)	Median	IQR (Range)
**Walking steps**						
*All*	7831.2	4412.5 (16415.8)	8620.26	4359.0 (15161.1)	7496.07	4493.58 (106.0–20828.3)
*Male*	7118.4	4456.3 (20686.3)	8209.29	3810.0 (1959.0–17652.8)	6874.15	4424.15 (142.0–20828.3)
*Female*	8676.7	4113.5 (17983.1)	9352.50	3702.5 (1441.5–18044.1)	8548.09	4123.02 (106.0–17376.2)
MWU-test	P < 0.001*		P = 0.51		P < 0.001*	
**Max. coherent distance (meters)**						
*All*	846.9	603.1 (6037.1)	995.66	504.8 (355.1–2453.4)	798.7	612.95 (1.0–6038.1)
*Male*	806.75	675.4 (4940.43)	996.00	578.8 (355.1–2301.1)	761.48	664.63 (5.0–4945.4)
*Female*	864.50	500.8 (6037.1)	913.38	463.6 (434.6–2453.4)	840.40	501.33 (1.0–6038.1)
MWU-test	P = 0.004*		P = 0.45		P = 0.004*	
**Total distance (meters)**						
*All*	5589.42	3089.8 (17551.9)	6463.19	2641.1 (1153.0–12532.4)	5206.81	3025.51 (8.0–17559.9)
*Male*	5251.00	3269.2 (17539.9)	6318.29	2800.5 (1517.0–12524.2)	4825.07	3314.18 (20.0–17559.9)
*Female*	6015.50	2826.3 (12959.7)	6880.25	2271.8 (1153.0–12532.4)	5782.69	2667.20 (8.0–12967.7)
MWU-test	P = 0.24		P = 0.32		P < 0.1	
**Walking speed (m/sec)**						
*All*	1.17	0.11 (0.71)	1.24	0.08 (1.05–1.50)	1.15	0.10 (0.93–1.64)
*Male*	1.18	0.12 (0.69)	1.25	0.09 (1.09–1.44)	1.16	0.10 (0.95–1.64)
*Female*	1.16	0.11 (0.57)	1.22	0.09 (1.05–1.50)	1.14	0.10 (0.93–1.45)
MWU-test	P = 0.018		P = 0.032		P = 0.019	

Abr.: IQR: Interquartile Range; MWU: Mann-Whitney-U-test, N: number of participants

* significant differences between men and women

**Fig 2:**
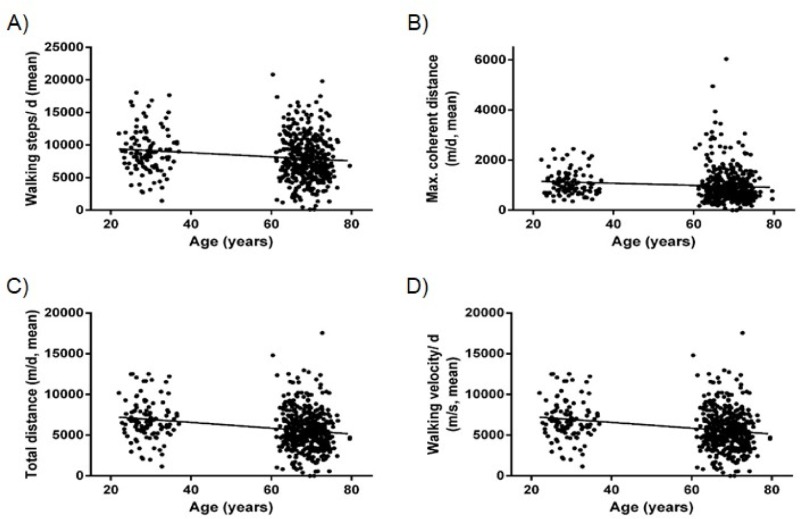
**A-D: Scatter plots of gait parameters** The figures show the gait parameters A) walking steps / day, B) maximum coherent distance / day, C) total distance / day, and D) average walking speed with respect to the participants’ age.

All analyzed walking parameters were correlated with age, as can be seen in Fig 2A–2D. Additionally, group comparisons using the Mann-Whitney-U-test and taking multiple testing into account (Bonferroni correction for 12 tests resulting in a p-value <0.0042 considered to be statistically significant) revealed significant differences in respect to the sex of the participants (Table 2). In the total cohort, the *number of walking steps per day* and the *maximal coherent distance* walked were significantly different between men and women. This difference was only observed in the older group when the two age groups were considered separately (Table 2).

We calculated a linear regression model for each of the four gait parameters (dependent variable) including sex, age, height and BMI as independent variables. The models showed independence of residuals as assessed by Durbin-Watson statistics with values between 1.9 and 2.1. All models were able to predict the respective dependent variable, as can be seen in Table 3.

**Table 3:** Model summary for the analyzed variables.

Dependent variable	F	Adj. R^2^	p
Walking steps	(3, 549) = 18.59	.113	< .001*
Max. coherent distance	(3, 549) = 4.84	.027	.001*
Max. distance	(3, 549) = 18.80	.114	< .001*
Gait speed	(3, 549) = 34.39	.195	< .001*

However, the models were only marginally able to predict the variance in the dependent variables, as reflected in the low R^2^-scores. In none of the models, all considered parameters were contributing significantly to the respective model. Age significantly contributed to predict the total distance walked per day (p = 0.005) and walking speed (WS) (p<0.001). Sex contributed significantly to the prediction of the number of walking steps per day (p = 0.03) and walking speed (p < .001), and BMI was able to contribute to the prediction of walking steps, the maximum coherent distance walked, and the total distance walked per day (all p<0.001). Further details can be derived from Table 4.

**Table 4:** Regression analysis for walking steps, maximum coherent distance, total distance and gait speed.

	Variable	*B*	*SE*_*B*_	*β*	p
**Walking steps (m)**	Intercept	13810.92	4537.07		
	sex	850.51	390.28	.122	.030*
	Age (years)	-10.13	9.08	-.050	.265
	Height cm)	-.744	22.01	-.002	.973
	BMI (kg/m^2^)	-236.20	34.97	-.287	< .001*
**Maximum coherent distance (m)**	Intercept	1026.07	889.25		
	sex	25.49	76.49	.020	.739
	Age (years)	-1.17	1.78	-.031	.512
	Height cm)	3.69	4.31	.052	.392
	BMI (kg/m^2^)	-24.80	6.86	-.161	< .001*
**Total distance (m)**	Intercept	10000.76	3364.94		
	sex	320.46	289.45	.062	.269
	Age (years)	-18.92	6.74	-.125	.005*
	Height cm)	4.78	16.32	.017	.770
	BMI (kg/m^2^)	-166.61	25.94	-.273	< .001*
**Gait speed (m/sec)**	Intercept	1.642	.117		
	sex	-0.35	.010	-0.185	.001*
	Age (years)	-0.03	.000	-.475	.000*
	Height cm)	-.002	.001	-.152	.006*
	BMI (kg/m^2^)	.000	.001	.015	.713

**Abr.:** *B*: unstandardized regression coefficient; *SE*_*B*_: Standard of the coefficient; *β*: standardized coefficient; p: statistical significance (* = significant contributor)

## Discussion

The results from our analysis demonstrate a clear inverse association between age and RWWS and the distance walked per day in a non-selected German population, thereby confirming the key finding by Schimpl et al. (2011) within a selected UK-based population [19].

While in the group of younger participants RWWS was comparable to the results obtained by Schimpl et al. (2011) [19], RWWS in the group of older people was lower. Several reasons might contribute to this observation. The cohort in the study by Schimpl et al. consisted of blood donors within the NHS Blood and Transplant Service (NHSBT). As such, blood donations have strict prerequisites in order to protect both the donor and the receiver of the blood sample. Consequently, any acute disease prohibits blood donation as well as many chronic conditions such as cardiovascular diseases, stroke, Mb. Parkinson or, in many cases, diabetes mellitus to name just a few. Additionally, older people cannot donate blood beyond the age of 70. Therefore, it can be assumed that recruitment of the older participants in the study by Schimpl et al. resulted in a cohort in whom the subgroup of older adults mainly was in a state of very good and as such even better health than the relatively healthy ambulatory participants of BASE-II. Furthermore, due to the recruitment process the number of participants was very low in this age group. Therefore, we assume that our results, generated employing a nearly identical measurement protocol with the identical type of sensor provide more representative data especially for older participants as well as a broader data basis for analyzing factors influencing RWWS in older adults. At the same time, the results for RWWS from Schimpl et al. regarding the younger cohort was verified by our analysis. For this reason, we think that the results of our analysis underline the general validity and applicability of sensor-based measurements of spatio-temporal gait parameters in the daily living environment of subjects of all age groups.

Our regression models revealed age as a significant contributor to the total distance covered per day and the RWWS of the participants. This is also in concordance with the findings by Schimpl et al. as well as other studies [27]. On the other hand, walking steps/day and the maximum coherent distance were not significantly associated with age in our cohort. Additionally, BMI was significantly associated with all gait parameters tested but WS, while height was only associated with RWWS. As reflected by the low adjusted R^2^ values, the variance of the dependent variables cannot be explained adequately by the applied regression models based on age, sex, height and BMI alone. Therefore, we assume that these gait parameters are influenced by several additional factors not adequately measured in our study. A variety of chronic diseases is discussed to negatively influence gait speed and walking ability; all with a positively associated prevalence with age. For example, chronic obstructive pulmonary disease [28] and lower kidney function [29] have been shown to be associated with lower gait performance. Similar associations were observed for other chronic diseases such as diabetes or arthritis, but also for low muscle mass as a defining parameter of sarcopenia [30]. These associations were independent of chronic diseases while a combination of both seemed to have the most profound negative impact on functional performance [30].

Most of these studies, however, focused on single measures, e.g. gait speed, and did not use long-term measurements of various gait parameters. Future studies therefore need to focus on the above mentioned and further potential factors to provide analytic models that are able to predict the consequences of changes in RWWS as well as other gait parameters. Furthermore, such models should be able to differentiate between individual factors that might be of clinical importance and environmental factors that can influence walking speed but do not reflect the individuals’ state of health.

Finally, sex contributed to the models predicting the number of walking steps as well as the RWWS in the current study. Direct comparisons between men and women in the total cohort using the Mann-Whitney-U-test showed significant differences for walking steps and the maximum coherent distance covered. When comparing men and women in both age groups, no such differences could be observed in the group of younger participants. In the older cohort, in contrast, women took significantly more walking steps per day and demonstrated a higher maximum coherent distance when compared to men. While this, again, suggests that more factors than age or sex contribute to changes and differences in RWWS, the nonetheless observable differences especially in the cohort of older adults command some consideration. Obviously, sex related differences tend to get larger with increasing age. In our cohort, women took more walking steps per day. While there was a significant difference only for all participants and older participants, the effect was observable as a trend in the younger cohort too and became more pronounced in the group of older adults. The same pattern was observed in all other evaluated parameters, although the direction of this was reversed in walking speed. Therefore, we conclude that sex-related differences exist in gait-related measurements that should be taken into account when interpreting results in future studies. However, as the mechanism behind the observed differences both for age and sex as of now remains largely unclear, the need to further examine these factors more deeply is obvious. As the sensor itself was tested against a gold standard in a comprehensive validation study [23], we deem the ability of the sensor to measure gait in real life from a purely technical standpoint as valid. On the other side, questions remain on how to interpret measurements of RWWS in older people. This includes which factors are contributing to a decline of RWWS-related parameters and how to use these factors in early detection of risk factors associated with older age. In this, some factors might contribute to gait characteristics gathered in real-life that have to be taken into account more thoroughly. One of the inclusion criteria for our analysis was that the actibelt sensor had to be worn at least 10 hours per day. We had, however, no exact information on the level of activity participants were exhibiting when the belt was not worn. So, it might be possible that the sensor was donned at either times of high activity or leisure time while keeping it off at other activities, leading to a bias of our overall results. While we can assume to a certain degree that such a potential bias has been evened out based on the size for our sample, especially the maximum coherent distance measured each day seems to be more prone to a measurement bias induced by the wearing times in comparison to the other parameters used in this analysis. In this, a potential analysis of wearing times would not have revealed the levels of activity during times the belt was not worn. For this reason, we did not pursue to include this factor in our analysis. Therefore, more detailed information on wearing times and accompanied activities are warranted in future studies.

The median RWWS calculated in our analysis is comparable to the gait speed values reported by Cesari et al. (2005) [4]. However, WS reported in other publications demonstrated a high variability on the obtained values. Samson et al. (2001) reported values of more than 1.3 m/sec in healthy older adults of 60 years or older [31]. In two systematic reviews reporting WS for older people in hospital settings or long-term care facilities, much slower WS values were reported. While Peel et al. (2012) calculated a mean WS of 0.58 m/sec for usual WS and 0.89 m/sec for maximal WS when pooling all WS data of the included studies [5], usual WS in the review conducted by Kuys et al (2014), the included studies exhibited between 0.14 and 0.92 m/sec [32]. Both studies reported a variety of measurement regimes with distances between 4 and 20 meters. Such variances could be explained by a study that could demonstrate how differences in testing protocols for measuring WS could lead to different results [33]. While this shows potential difficulties in comparing scientific results on WS, it also shows how situational changes and the setting in which the measurements take place can affect results of walking and mobility assessments. In another study, different focusing strategies for detecting obstacles during walking tasks could be observed in fallers and non-fallers [34]. Obstacle recognition and deriving appropriate strategies to avoid them can interfere with activity-related goals in daily life and thus can influence the overall walking speed in the real-life environment of older people. All these factors can contribute to a lower RWWS in older adults who are capable, from a purely functional and physiological standpoint, to walk at a faster pace. This is also illustrated by a recent analysis of BASE-II data, where we found the subjective age of participants well correlated with the results of mobility measurements in a laboratory setting, but not with RWWS measured in daily life [35]. Other potential factors influencing walking speed can be slippery or unstable surfaces that make an adaption of the walking strategy necessary [36,37] or exhibit effects on walking speed over time, e.g. getting slower after a certain time or distance traveled due to pain or exhaustion. Again, the additional impact of chronic diseases like diabetes or peripheral arterial occlusive disease on the ability to adapt to external and situational factors such as changes in ground surface have to be considered as well.

In our estimation and expectation, these situational factors will play a significant role when WS is measured over a longer period of time and within the living environment of the participants. It can be expected that measuring RWWS with a body worn sensor will measure WS in many different situations of everyday life and thus encompasses many factors influencing WS in both directions. Thus, the RWWS could be a much more realistic parameter for evaluating the walking ability of older people than laboratory conditions. It can be assumed that this kind of influence can be found for other gait-related parameters as well.

Therefore, an urgent demand for gait-related research is warranted where standard measurements of gait in laboratory settings are compared to real life and long-term measurements. Ideally, such comparisons would take into account different situations in which external factors have an influence on walking speed and other mobility parameters. This would enable researchers to take a more in-depth look at how walking as a physical function and an everyday ability represents different dimensions of mobility and, subsequently, how measurements of either kind can be used to derive more precise and specific implications for risk stratification, physical therapy, prevention and rehabilitation; and other fields of patient care of older people.

## Conclusion

Both age and sex contribute to differences in the absolute values of gait-related parameters. Therefore, any interpretation and clinical decisions based on the results of gait measurements have to take into account both these variables. In order to be able to derive valid conclusions, normative values for meaningful differences in RWWS measurements with respect to age and sex have to be established. Such normative values may enhance the value of absolute values of RWWS measurements in prediction of functional decline as well as the onset of age-related chronic conditions in older people.

## Supporting information

S1 Table Sociodemographic variables of the BASE-II cohort medical part at baseline (N = 2.171). (PDF)
