# Correction: Long-term gait measurements in daily life: Results from the Berlin Aging Study II (BASE-II)

**DOI:** 10.1371/journal.pone.0233430

**Published:** 2020-05-13

**Authors:** Jörn Kiselev, Timur Nuritdinow, Dominik Spira, Nikolaus Buchmann, Elisabeth Steinhagen-Thiessen, Christian Lederer, Martin Daumer, Ilja Demuth

In Fig 2, Panel C is incorrectly duplicated in Panel D. Please see the correct Fig 2 here.

**Fig 2:**
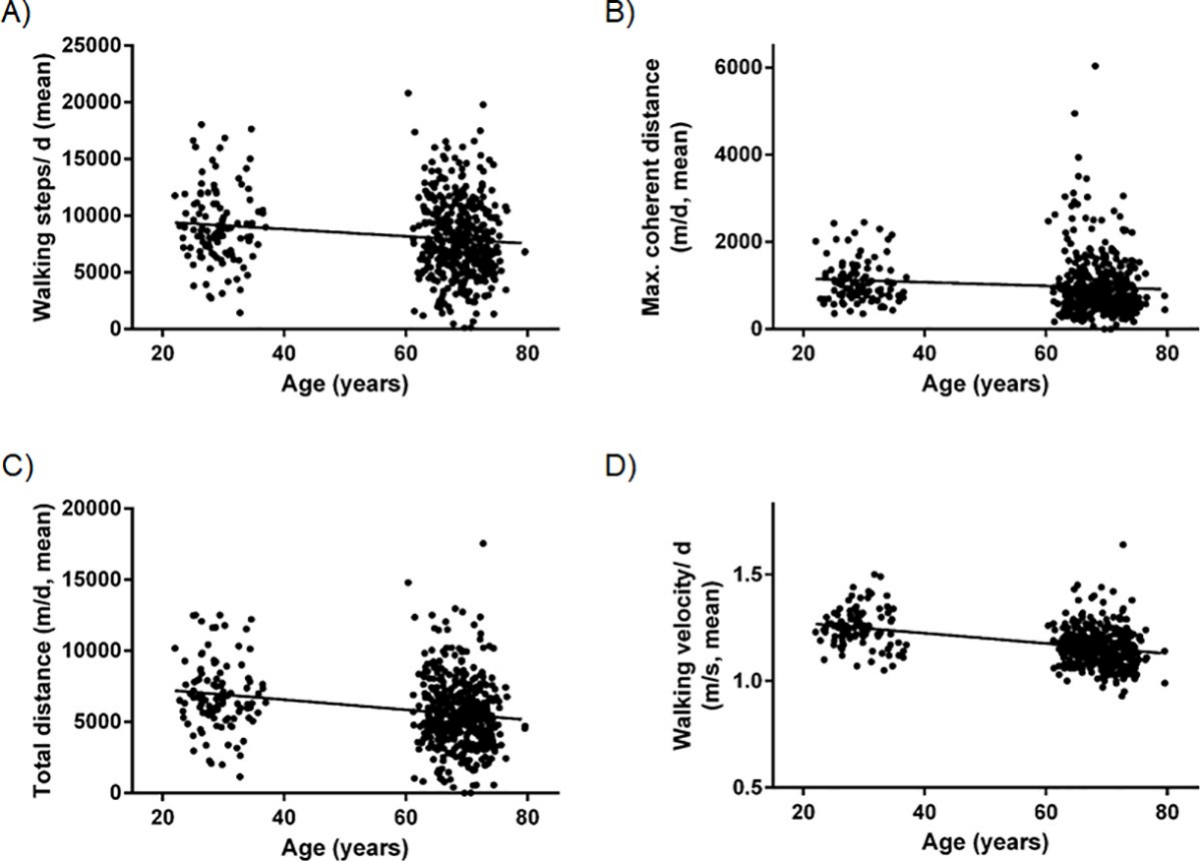
**A-D: Scatter plots of gait parameters** The figures show the gait parameters A) walking steps / day, B) maximum coherent distance / day, C) total distance / day, and D) average walking speed with respect to the participants’ age.
