# Supplementary material for: Long-term gait measurements in daily life: Results from the Berlin Aging Study II (BASE-II)
Source: PLoS One. 2019 Dec 11;14(12):e0225026. doi: 10.1371/journal.pone.0225026 (PMC6905575; doi:10.1371/journal.pone.0225026)
Supplement: S1 Table — (PDF) [file pone.0225026.s001.pdf]

**Table 1: Sociodemographic variables of the BASE-II cohort. medical part. at baseline (N= 2.171)**

| Variable | All participants | | Younger participants | | Older participants | |
|---|---|---|---|---|---|---|
| | **N** | **%** | **N** | **%** | **N** | **%** |
| **Sex** | | | | | | |
| *Male* | 1.044 | 48.1 | 235 | 47 | 809 | 48.4 |
| *Female* | 1.127 | 51.9 | 265 | 53 | 862 | 51.6 |
| | **Median** | **IQR (Range)** | **Median** | **IQR (Range)** | **Median** | **IQR (Range)** |
| **Age (years)** | | | | | | |
| *All* | 67.2 | 7.9 (22.0-84.6) | 28.8 | 4.7 (22.0-37.0) | 68.7 | 5.3 (60.2-84.6) |
| *Male* | 67.6 | 8.5 (22.0-82.8) | 29.3 | 4.6 (22.0-37.0) | 69.1 | 5.5 (60.2-82.8) |
| *Female* | 66.8 | 7.4 (22.7-84.6) | 28.5 | 4.6 (22.7-36.5) | 68.4 | 5.0 (61.3-84.6) |
| **Height (cm)** | | | | | | |
| *All* | 170[1] | 13.5(144-199.7)[1] | 173.9[2] | 12.1 (152.2-199.7) | 169.0[3] | 13.3 (144-197) |
| *Male* | 176.6[4] | 9.0 (155.3-199.7) | 180.1[5] | 8.7 (155.3-199.7) | 175.5[6] | 8.7 (156.3-197) |
| *Female* | 164[7] | 9.1 (144.0-190.0) | 169.0 [8] | 7.9 (152.2-190.0) | 163.0[9] | 8.1 (144-182.5) |
| **BMI** | | | | | | |
| *All* | 25.5[10] | 5.5 (11.6-47.7) | 22.4[11] | 4.8 (11.6-43.9) | 26.4[12] | 5.2 (17-47.7) |
| *Male* | 26.1[13] | 4.6 (16.6-44.2) | 23.5[14] | 4.3 (16.6-43.9) | 26.9[15] | 4.3 (19.1-44.2) |
| *Female* | 24.7[16] | 6.1 (11.6-47.7) | 21.6[17] | 4.3 (11.6-40.5) | 25.7[18] | 5.9 (17-47.7) |

Abr.: BMI: Body Mass Index; IQR: Interquartile Range; N: number of participants

[1] 46 missings. [2] 13 missings. [3] 33 missings. [4] 18 missings. [5] 7 missings. [6] 11 missings. [7] 28 missings. [8] 6 missings. [9] 22 missings. [10] 46 missings. [11] 13 missings. [12] 33 missings. [13] 18 missings. [14] 7 missings. [15] 11 missings. [16] 28 missings. [17] 6 missings. [18] 22 missings
